# Vitamin D and estrogen steroid hormones and their immunogenetic roles in Infectious respiratory (TB and COVID-19) diseases

**DOI:** 10.1590/1415-4757-GMB-2022-0158

**Published:** 2023-02-06

**Authors:** Maria Eduarda de Albuquerque Borborema, Thays Maria Costa de Lucena, Jaqueline de Azevêdo Silva

**Affiliations:** 1 Universidade Federal de Pernambuco, Departamento de Genética, Laboratório de Genética e Biologia Molecular Humana (LGBMH), Recife, PE, Brazil.; 2 Universidade Federal de Pernambuco, Laboratório de Imunopatologia Keizo Asami (LIKA), Recife, PE, Brazil.

**Keywords:** Tuberculosis, COVID-19, Vitamin D3, 17β-estradiol, Mycobacterium tuberculosis, SARS-CoV-2

## Abstract

The role of steroid hormones against infectious diseases has been extensively studied. From immunomodulatory action to direct inhibition of microorganism growth, hormones D_3_ (VD_3_) and 17β-estradiol (E_2_), and the genetic pathways modulated by them, are key targets for a better understanding pathogenesis of infectious respiratory diseases (IRD) such as tuberculosis (TB) and the coronavirus disease-19 (COVID-19). Currently, the world faces two major public health problems, the outbreak of COVID-19, accounting for more than 6 million so far, and TB, more than 1 million deaths per year. Both, although resulting from different pathogens, the *Mtb* and the *SARS-CoV-2*, respectively, are considered serious and epidemic. TB and COVID-19 present similar infection rates between men and women, however the number of complications and deaths resulting from the two infections is higher in men when compared to women in childbearing age, which may indicate a role of the sex hormone E_2_ in the context of these diseases. E_2_ and VD_3_ act upon key gene pathways as important immunomodulatory players and supporting molecules in IRDs. This review summarizes the main roles of these hormones (VD_3_ and E_2_) in modulating immune and inflammatory responses and their relationship with TB and COVID-19.

## Introduction

Steroid hormones have been the target of recent studies due to their immunomodulatory role in inflammation, in cardiovascular, autoimmune and in infectious diseases (Chocano-Bedoya and Ronnenberg, 2009; Lopes Marques, 2010; Kovats, 2015; Trenti *et al*., 2018). The main female hormone, 17β-estradiol (E_2_), present high circulating levels in women in childbearing age but its production declines considerably after menopause, usually when women become more susceptible to cardiovascular and infectious diseases. In fact, E_2,_ presents a wide and potent signalling among immune system cells towards a pro-inflammatory response, making women more prone in restraining infectious diseases during childbearing ages (Deguchi *et al*., 2001; Carr, 2003; Patel *et al*., 2018; Kadel and Kovats, 2018). 

Cholecalciferol or vitamin D_3_ (VD_3_) plays a key role in bone metabolism and calcium homeostasis, however, this hormone is capable of exerting extra-skeletal activities such as in cellular physiology, antiproliferative effects on cancer cells (Trochoutsou *et al*., 2015), modulation of the immune response and control of inflammation, roles performed through the binding to the Vitamin D Receptor (VDR) (Sassi *et al*., 2018). The functions performed by VD_3_ can be mediated by the transcriptional role of the VDR in the cell nucleus; and by a non-genomic mechanism, when the VDR induces rapid signalling by sensitising cell membrane and/or cytoplasmic proteins. Importantly, VD deficiency affects more than 1 billion people in the world and has been associated with a series of pathologies including infectious, autoimmune, and allergic diseases (Trochoutsou *et al.*, 2015).

Lower respiratory infections are among the three leading causes of death and disability in children and adults, and remain as the world’s most deadly communicable diseases, ranked as the 4th leading cause of death (World Health Organization, 2020). Over the last decades, epidemics of respiratory diseases caused by pathogens, whether viruses or bacteria, have been a major public health problem, especially tuberculosis (TB), which has one of the highest global mortality rates, and more recently the pandemic caused by the new coronavirus, Coronavirus Disease-19 (COVID-19), responsible for more than six million deaths worldwide (World Health Organization, 2022).

COVID-19 and TB are communicable diseases and epidemiological data on the profile of the infected population, including complication rates and deaths, have been collected worldwide. Although infection rates in these diseases are similar between men and women, it is observed that sex, age and genetic profile may influence the clinical outcome of these diseases, once women in childbearing age, compared to men in the same age group, have lower rates of infection complications and death in both diseases (Hertz and Schneider, 2019; Ahmed and Dumanski, 2020).

The steroid hormones VD_3_ and E_2_ share not only chemical similarities but also actions. They are able to act upon gene expression and mRNA stability in different tissues and defence cells from the innate and adaptive immune system allowing them to influence their response efficiency (Ing, 2005; Haussler *et al*., 2011). Emerging evidence supports the hypothesis that VD_3_ improves immunity by maintaining a balanced immune response. This receptor is expressed by most cells of the defence system such as T and B lymphocytes and antigen-presenting cells (APCs) (Trochoutsou *et al*., 2015). The VD_3_/VDR complex triggers the differentiation of innate cells such as monocytes into macrophages and in the adaptive response by modulating T cell differentiation (Durrant *et al*., 2022). VD_3_ produced locally by monocytes and macrophages can induce the expression of cathelicidins and β-defensin 2, which play a direct role in the response against pathogens as *Mycobacterium tuberculosis* (*Mtb*) (Ao *et al*., 2021).

E_2_ through Estrogen Receptors (ERs) can regulate cells and pathways in the innate and adaptive immune responses, as well as immune cell differentiation. ERs are ligand-dependent transcription factors and play key roles in mediation of long-range chromatin interactions and the formation of complexes that regulates gene expression by binding to estrogen response elements (EREs). ERs also participate in membrane-initiated steroid signalling to generate rapid responses. E_2_/ER activity shows dose- and context-dependent effects on innate immune signalling pathways (Kovats, 2015).

In this way, understanding the main pathways of action of these hormones against infections caused by pathogens can be an important weapon in the investigation of possible molecules that help in the improvement of the host’s immune response as well as in a containment of inflammation in these individuals in the response against these pathogens.

## Respiratory diseases

Respiratory tract diseases (RTDs) are pathological conditions that affect organs and tissues responsible for breathing (Forum of International Respiratory Societies, 2017). The respiratory system turns out to be the internal set of organs most susceptible to infections and injuries from the environment due to its constant exposure to particles such as chemicals and pathogenic microorganisms (Lee, 2017). Respiratory diseases include acute respiratory infections as well as chronic respiratory diseases such as asthma, chronic obstructive pulmonary disease, and lung cancer (Forum of International Respiratory Societies, 2017).

RTDs can be classified in different ways, from the organ or tissue affected to the pattern of signs and symptoms associated with the disease. They can range from mild, such as a simple cold, to serious illnesses such as bacterial pneumonia, TB, pulmonary embolism, lung cancer, asthma, and severe acute respiratory syndromes (Nair *et al*., 2011; Herefordshire Council, 2022).

Lower respiratory tract infections are among the top three causes of disability and death in children and adults. It is estimated that these infections cause nearly 4 million deaths per year. Moreover, acute infections in the lower respiratory tract in children increase the predisposition for chronic respiratory diseases development in adult life (Forum of International Respiratory Societies, 2017).

Infections can affect any part of the respiratory system, traditionally divided into upper respiratory tract infections (URTIs) and lower respiratory tract infections (LRTIs). The most common type of URTI is the cold, however, infections in specific organs such as sinusitis, tonsillitis, pharyngitis, and laryngitis are also frequent. In the lower respiratory tract, the most frequent disease is pneumonia, a pulmonary infection caused by different pathogens, from bacteria such as *Streptococcus pneumoniae* to *Mtb* that causes TB (Lozano *et al*., 2012; Wang *et al*., 2016; Lee, 2017).

TB has one of the highest global mortality rates, leading about 17% of infected individuals to death, which approximately a quarter are co-infected with the human immunodeficiency virus (HIV). This makes it the leading disease caused by a single infectious agent and one of the leading causes of death worldwide from infectious diseases (World Health Organization, 2021).

Lately, new pathogens that cause respiratory infections have emerged. In 2003, severe acute respiratory syndrome (SARS), caused by the Coronavirus (*SARS-CoV)*, a previously unknown pathogen, spread rapidly around the world. In 2019, a new coronavirus (*SARS-CoV-2*) triggered a pandemic that by September 2022 reached the mark of more than 615 million infected and 6.5 million deaths worldwide (World Health Organization, 2022).

### Tuberculosis

TB is a notifiable infectious disease caused by *Mtb*. Its transmission is almost exclusively by inhalation of aerosols, containing the bacillus, released during the cough of infected individuals. Usually affects the lungs (pulmonary TB), but can affect other organs such as bones, liver, intestines, and lymph nodes (extrapulmonary TB) (Dheda *et al*., 2016). 

According to WHO, in 2020 a large global drop in the number of people newly diagnosed with TB was observed, from 7.1 million in 2019 to 5.8 million in 2020 (18% of reduction) (World Health Organization, 2021). This decrease may be a result of the likely underreporting of the number of TB cases due to the COVID-19 pandemic. Another consequence of the pandemic was the reduction in TB treatment access, which is reflected in the increase of TB deaths (1.3 million in 2020) (World Health Organization, 2021).

It is known that TB affects both males and females in all age groups, but a higher incidence is observed in men over 15 years of age. In 2020, men accounted for 56% of all TB cases, while women of the same age group accounted for 33% of cases (World Health Organization, 2021). Brazil is now the centre of WHO concern in Americas, where TB incidence appears to be increasing owing to an upward trend. In 2020, Brazil recorded 66,819 new TB cases with an incidence coefficient of 31.6 cases per 100,000 habitants. Following the global trend, between 2011 and 2020, 69.0% of new TB cases were male (Secretaria de Vigilância em Saúde, 2021).

A relatively small portion, 5-10% of the nearly 2 billion people infected with *Mtb* have developed or will develop active TB during their lifetime. However, the likelihood of developing the disease is much higher among patients co-infected with HIV or risk factors such as malnutrition, diabetes, smoking and excessive alcohol consumption (World Health Organization, 2019).

The development of the TB active form depends on several factors from lifestyle to immunological status, comorbidities like diabetes and acquired immunodeficiency syndrome (AIDS) as well as the genetic profile of the infected individual (Menon *et al*., 2016; Mathema *et al*., 2017).

TB transmission occurs when the individual inhales the infectious nuclei and the *Mtb* manages to cross the URTIs and the bronchi, to reach the pulmonary alveoli only then where it settles and is phagocytosed by cells of the immune system, especially alveolar macrophage (Ketata *et al*., 2015).

Immunocompetent individuals can control the development of TB through two specific lines of defence: the first one formed by phagocytic cells, such as polymorphonuclear cells, monocytes, and alveolar macrophages, thus constituting the innate immune response. And the second one, characterised by an acquired immune response (Gupta *et al*., 2018; Furin *et al*., 2019). 

Once *Mtb* bacilli are phagocytosed by alveolar macrophages, occurs the internalisation into the phagosome, which fuses with the lysosome, forming the phagolysosome, where a series of granules and other toxic products produced by macrophages and stored in the lysosome are released. The lysosome contains numerous hydrolytic enzymes and has an acidic content. Phagolysosome formation is considered to be the primary infection control mechanism and occurs via interferon-γ (IFN-γ) (Fogel, 2015).

After phagocytosis, the inhaled bacilli remain in cytoplasmic vacuoles and are presented to CD4+ T lymphocytes by the major histocompatibility complex class II (MHC-II), present in macrophages, dendritic cells (DCs), and B lymphocytes. These cells are APCs and produce inflammatory cytokines such as tumor necrosis factor (TNF) and interleukin-1 (IL-1), capable of recruiting neutrophils and monocytes maintaining the innate response (Gupta *et al*., 2018; Furin *et al*., 2019). Macrophages also activate a specific immune response against TB, when infected, they release IL-12 and IL-18, which predominantly stimulate CD4+ T lymphocytes to release IFN-γ, which in turn stimulates *Mtb* phagocytosis (Cliff *et al*., 2015; Gupta *et al.*, 2018). However, if the bacilli are not killed during this initial interaction, they can proliferate within DCs and alveolar macrophages at a high rate of growth (Fogel, 2015; Domingo-Gonzalez *et al*., 2016).

The acquired immune response is mediated by pathogen-recognition receptors (PRRs) that are expressed by these cells and recognize pathogen-associated molecular patterns (PAMPs) expressed by the *Mtb* (Hossain and Norazmi, 2013). Among these receptors several stand out in triggering the inflammatory response against *Mtb*, including nucleotide oligomerization NOD-like receptors (NLRs) and toll-like receptors (TLRs). The purpose of *Mtb* presentation to these receptors is the induction of various intracellular signalling cascades for cytokine production, whether pro- or anti-inflammatory, thus regulating the inflammatory process (Fogel, 2015).

Recognition of specific microbial ligands by TLRs activates inflammatory signalling pathways (Apt *et al*., 2017). Among the main TLRs that recognize *Mtb* are TLR2, TLR9 and TLR4 (Means *et al*., 1999). Activation of these receptors on the surface of macrophages initiates the recruitment of various adapter proteins such as the myeloid differentiation primary response gene 88 (MyD88). In sequence, the activation of a series of molecules of the signalling pathway occurs, culminating in the translocation of nuclear factor- κB (NF-κB) to the nucleus and in the activation of the transcription of immune response genes, such as cytokines, chemokines and enzyme induced nitric oxide synthase (iNOS) (Apt *et al.*, 2017).

Mycobacterial recognition by TLRs activates the transcription of genes encoding the NF-κB and the iNOS by the secretion of TNF, responsible for high levels of nitric oxide (NO). NO production is strongly associated with *Mtb* resistance because reactive nitrogen intermediates (RNI) are toxic to *Mtb*, and infection is exacerbated by enzyme nitric oxide synthase 2 (NOS2) inhibition (Sia and Rengarajan, 2019). It is also known that NO production after IFN-γ signalling limits inflammation by inhibiting IL-1β processing by the inflammasome (Mishra *et al*., 2013).

The recognition of *Mtb* by TLRs, in fact, induces a predominantly pro-inflammatory response. However, signalling via TLR2 also increases the secretion of IL-10, an anti-inflammatory T helper-2 (Th2) cytokine, by DCs and macrophages, suggesting a pathogen defence mechanism in controlling the host’s inflammatory response (Salgame, 2005).

The initial infection is dominated by a Th1 immune response, however, if the infection is not contained, a gradual shift to Th2 response occurs (Moutinho, 2011). Several studies try to clarify the immunological mechanisms in infections by intracellular bacteria, and the finding of cytokines produced by Th2 cells, such as IL-4 and IL-10, may reflect the inability to respond to these bacteria. These and other cytokines that suppress Th1 activity and markers of Th2 activity, such as immunoglobulins (Ig) E and IgG4, are frequently found in advanced TB (Ferraz *et al*., 2006). 

The balance between Th1 cytokines to inhibit mycobacterial growth (IFN-γ and TNF), or Th2 to accelerate *Mtb* growth (IL-4 and IL-10), may be the key in the regulation of mycobactericidal activity in infected macrophages (Ferraz *et al*., 2006). Alveolar cells from patients with active pulmonary TB express Th1 cytokines (IL-2 and IFN-γ) in an environment also known as Th2 due to the presence of IL-10 and IL-4. It is not the increase in Th1 cytokines, but the increase in Th2 cytokines, which play an important role in the TB progression (Novikov *et al*., 2011). 

However, the immune response against the *Mtb* should not be interpreted solely based on a Th1/Th2 regulatory balance. The successful elimination of *Mtb* depends on the correct interaction between the innate and the acquired response, in which numerous cells and a wide network of chemical mediators participate. Although TB is an infectious disease, the clinical outcome (latent TB or TB disease) varies by the influence of other factors such as immunity and the genetic background of the host (Malik and Schurr, 2002).

Many of the studies that address the genetics of the host in the face of *Mtb* infection are based on the association between the development of TB and the variation in the frequencies of polymorphisms in candidate genes. Several studies have been associating genetic factors with susceptibility or protection against *Mtb* infection (Chocano-Bedoya and Ronnenberg, 2009; Chen *et al*., 2013; Aravindan, 2019). In the immune response against the *Mtb*, allele and genotype frequencies from inflammatory gene polymorphisms (*IFN-γ*, *IL-10*, *TNF*, *TLR4* and *VDR*) vary considerably across populations (Malik and Schurr, 2002; Chen *et al.*, 2013; Simmons *et al*., 2018; Aravindan, 2019; de Albuquerque Borborema *et al*., 2020; Chen *et al.*, 2021). Polymorphisms that cause alterations on cytokine serum levels (IFN-γ, TNF and IL-17) are in association with a worse prognosis and a higher mortality rate (Zhang *et al*., 2011; Simmons *et al.*, 2018).

Variants in the *TLR* pathway genes are can deregulate the cellular immune response and may influence the susceptibility to the bacillus (Berrington and Hawn, 2007). Polymorphisms in the *TLR2* signalling domain (G2258A (R753Q)) can alter the response to stimulation with *Mtb* lipoproteins, altering the response triggered by NF-κB (Schröder *et al*., 2005). Other polymorphisms such as the GT dinucleotide repeat in intron II of *TLR2* are associated with increased susceptibility to TB, as well as altered activity of the *TLR2* promoter (Yim *et al*., 2006).

DCs recognize *Mtb* through receptors such as TLR1, TLR2, TLR6, TLR9, NOD2, DC-SIGN and possibly TLR4 and Dectin-1. Several adaptive immune responses can be activated depending on the activated receptor (Arentz and Hawn, 2007). Host genetic variations can influence the adaptive immune response to *Mtb* triggered by DCs. Some alterations are described in the *MHC* gene and associated with *Mtb* susceptibility (Fernando and Britton, 2006). Changes in the DC-SIGN encoded by *CD209* gene at the promoter region (-871G and -336A) are associated with TB susceptibility, although reports are still conflicting (Barreiro *et al*., 2006; Gómez *et al*., 2006). The homozygous mutation in the IL-12p40 subunit, which inhibits the production of IL-12, is associated with primary immunodeficiency and increased susceptibility to TB (Casanova and Abel, 2002). 

The outcome of infection with *Mtb* depends on several factors, among them the effective acidification of the phagosome in the phagosome-lysosome fusion, the activation of the inflammasome complex by NLRs, that results in the release of IL-1β and IL-18 (Gröschel *et al*., 2016), and the upregulation of host genes that are required for activation of intracellular death mechanisms such as reactive oxygen species (ROS), NO, the production and release of the antimicrobial cathelicidin peptide (CAMP) and, finally, the activation of pathways of cell death by the host (pyroptosis, apoptosis and necrosis) (Behar *et al*., 2010; Verrall *et al*., 2014).

All these mechanisms are activated by genomic or non-genomic pathways. Both share their regulation through several mechanisms, including the modulation performed by steroid hormones such as VD_3_ and E_2_.

### COVID-19

In early December 2019, the outbreak of a new respiratory disease began, COVID-19, caused by *Severe Acute Respiratory Syndrome Coronavirus-2* (*SARS-CoV-2*), in Wuhan City, Hubei Province, China, and spread around the world causing the biggest pandemic of the 21st century (Harapan *et al*., 2020).

Daily, since the beginning of the COVID-19 pandemic, the WHO updates the number of new cases and deaths resulting from COVID-19. As of September 26, 2022, 612.236.677 cases have been reported worldwide. As for the number of deaths, 6.514.397 have been recorded since December 2019 (World Health Organization, 2022).

According to WHO, there is a little difference in the reported numbers of cases in men and in women. Among over 700,000 confirmed cases reported by April 2020, the sex rate is almost equal Male: Female cases = 1.03:1. However, this sex ratio varies with age: the younger (20-29 years old) and older (80 years and older) age groups, present more cases in women than men. On the other hand, for age groups 0-9 years, 60-69 years and 70-79 years, there are more cases in men than women (Global Health 50/50, 2022). According to global data for every ten women, there are twelve hospitalisations, 17 intensive care unit (ICU) admissions and 13 male deaths by COVID-19 and its complications. These data show that although men and women have the same rate of infection, men generally complicate and die more compared to women. 

*SARS-CoV-2* is the seventh member of the family of coronaviruses that infect humans (Cheng and Shan, 2020) and is considered a new RNA virus, belonging to the order *Nidovirales*, to the genus *Betacoronavirus,* subgenus *Sarbecovirus*, of the family *Coronaviridae* and subfamily *Orthocoronavirinae* (Chen *et al.*, 2020; Lu *et al*., 2020; Paraskevis *et al*., 2020). 

Coronaviruses have a positive-sense, non-segmented, single-stranded RNA genome of about 30 kilobases (kb). *SARS-CoV-2* genome contains two untranslated regions (UTRs): the 5’-cap structure and the 3’-poly-A tail, plus a single origin of replication (ORF) encoding a polyprotein (Chen *et al*., 2020; Lu *et al*., 2020). Its genome has 29,891 base pairs (bp) in length with a GC content of 38% (Dhama *et al*., 2020). The following sequence follows in the 5’-3’ direction: ORF1a, ORF1b and structural protein genes - Spike (S), Envelope (E), Membrane (M) and Nucleocapsid (N). Some accessory protein genes, such as ORF3a, 7 and 8, are inserted into structural protein genes (Chan *et al*., 2020; Chen *et al.*, 2020; Lu *et al.*, 2020; Paraskevis *et al*., 2020; Wu *et al*., 2020).

*SARS-CoV-2* infection is transmitted through large droplets generated during the coughing and sneezing of patients, but it can also occur in asymptomatic people or before the onset of symptoms by droplets expelled during speech (Rothe *et al*., 2020). Binding to a receptor expressed by host cells is the first step of viral infection, followed by fusion of the virus with the cell membrane (Rothan and Byrareddy, 2020). It is known that type II pneumocytes are the main target of the virus. The infection occurs initially by the binding of the receptor binding domain (RBD) of the S protein of the virus and the cellular receptor that has been identified as the angiotensin-converting enzyme 2 (ACE2) receptor (Jaimes *et al*., 2020; Wan Y *et al*., 2020). ACE2 is expressed in type I and II alveolar epithelial cells. Men have a higher level of ACE2 in pneumocytes compared to women. The expression of ACE2 is autoregulated by the binding of ACE2 to *SARS-CoV-2*, which can lead to alveolar cell damage by increasing the expression of this receptor. This damage triggers a series of systemic reactions and even death (Sun *et al*., 2020). 

As the *SARS-CoV-2* entry receptor into the host cell, ACE2, is highly expressed by the epithelial cells (pneumocytes) of the alveolar space on the apical side of the lungs, this virus can easily enter and destroy this tissue. Receptor recognition is not the only determinant for successful infection, immediately after binding to ACE2, *SARS-CoV-2* enters the host cell and triggers the activation of an innate immune response. In order to stay alive and replicate in the host, the virus has the ability to inhibit or avoid the signalling of innate host response (Hamming *et al*., 2004; Jia *et al*., 2005; Yuki *et al*., 2020). 

The host immune system response to viral infection by mediating inflammation and cellular antiviral activity are critical to inhibiting viral replication and spread. However, the exacerbated immune response in conjunction with the lytic effect of the virus on host cells results in pathogenesis (Harapan *et al*., 2020). As a first step, immune cells detect the viral infection by identifying PAMPs such as viral RNA. These PAMPs are able to bind and activate PRRs such as TLR3, TLR7, cytoplasmic RNA sensors (retinoic acid-inducible gene, RIG-I) and melanone differentiation-associated protein (MDA5) in immune cells, which results in the activation of these cells (Felsenstein *et al*., 2020).

Normally, activation of TLR3 and TLR7 results in nuclear translocation of transcription factors NF-κB and interferon regulatory factor 3 (IRF3), while activation by RIG-I and MDA5 results in activation of IRF3. This activation triggers the expression of IFN-I through IRF3 and other innate pro-inflammatory cytokines (IL-1, IL-6, TNF) through NF-κB (de Wit *et al*., 2016; Prompetchara *et al*., 2020). In this context, IFN-I and other innate pro-inflammatory cytokines promote their own expression through self-amplification, where IFN-I activates the IFN-α receptor complex (IFNAR) that results in phosphorylation/activation of transcription factors 1 and 2 of the signal transducers and activators of transcription (STAT) family, while the activation of IL-1, IL-6 and TNF receptors fuels the expression of pro-inflammatory cytokines through NF-κB (de Wit *et al.*, 2016; Lazear *et al*., 2019). Activation of the innate response and initiation of the adaptive response should result in pathogen elimination and host recovery (Felsenstein *et al*., 2020). 

Suppression of innate immune response mechanisms in infected epithelial cells and infected monocytes and macrophages, allows *SARS-CoV-2* to proliferate without triggering the innate antiviral response machinery. However, at a later stage of infection, infected cells undergo cell death and release viral particles along with intracellular components that trigger inflammatory mechanisms of the innate immune response through their recognition by PRRs of immune system cells such as DCs and macrophages. As a result of the activation of the innate immune response and consequent expression of pro-inflammatory cytokines such as IL-1β, IL-6 and TNF, immune cells of the adaptive response, such as T and B lymphocytes, engage in host defence against the virus (Felsenstein *et al*., 2020). 

*SARS-CoV-2* can also escape, even partially, from the adaptive response by inducing T cell apoptosis (Yi *et al*., 2020). However, lymphocyte depletion can occur due to the over-expression of pro-inflammatory cytokines by innate (uninfected) immune cells that are recruited to the lungs where they trigger hyper-inflammation, known as a *Cytokine Storm* (Costela-Ruiz *et al*., 2020). 

Circulating monocytes respond to granulocyte-macrophage colony-stimulating factor (GM-CSF) released by activated T cells. Subsets of CD14+ CD16+ inflammatory monocytes, which rarely exist in healthy controls, were found in large amounts in COVID-19 patients. These cells express high amounts of IL-6, which likely can accelerate the progression of the systemic inflammatory response (Yuki *et al*., 2020). Uninfected monocytes/macrophages and neutrophils recruited to the site of infection exhibit strong and poorly controlled inflammatory responses, resulting in tissue damage and systemic inflammation, which contribute to host morbidity and mortality (Zhang *et al*., 2020).

Another factor that contributes to organ damage and insufficient results against infection is the early production of neutralising antibodies against *SARS-CoV-2*. The antibody-dependent enhancement (ADE) phenomenon contributes to the accumulation of damage during viral infection, which promotes cellular uptake of viral particles bound to immune complexes through their binding to Fcγ receptors (FcγR). This can contribute to persistent viral replication in immune cells, including infected APCs, but also to immune complex-mediated inflammatory responses, which contribute to tissue and organ damage (Takada and Kawaoka, 2003; Jin *et al*., 2020; Fu *et al*., 2020).

## Steroid hormones

Steroid hormones are responsible for regulating several physiological and developmental mechanisms during life. They essentially orchestrate basal cell, tissue and organ functioning and when they are impaired or deficient several organs and tissue may collapse leading deleterious effects and disease establishment. Their action mechanism is mainly through cytosolic receptors that complexes with homo or hetero-dimers and follows to the nucleus whereas binds to response elements (RE) in DNA and activate or repress gene transcription (Schwartz *et al*., 2016).

Steroid hormones are lipid in nature, they have a basic core that is derived from the cholesterol molecule. Its synthesis occurs in some tissues such as the cortex of the adrenal glands and gonads, which express different forms of the P450 enzyme complex (Shahidi, 2001). They are classified as corticosteroids, androgens, estrogen and progestins being responsible for several functions in the body from metabolic, hydro-saline, and sexual control (Schwartz *et al*., 2016).

In this review we will address two of the main steroid hormones with immunoregulatory action and recently described with activity against pathogens: Vitamin D_3_ and 17β-estradiol.

### Vitamin D

Vitamin D (VD) represents the nomenclature for a group of fat-soluble secosteroid hormones responsible for increasing intestinal calcium absorption, in addition to several other biological effects (Holick, 2006). In humans, the most important compounds in this group are: 25-hydroxyvitamin D_2_ or ergocalciferol (VD_2_) and 1,25α-dihydroxyvitamin D_3_ or cholecalciferol (VD_3_) (Al-Zohily *et al*., 2020). 

**Figure 1 -:**
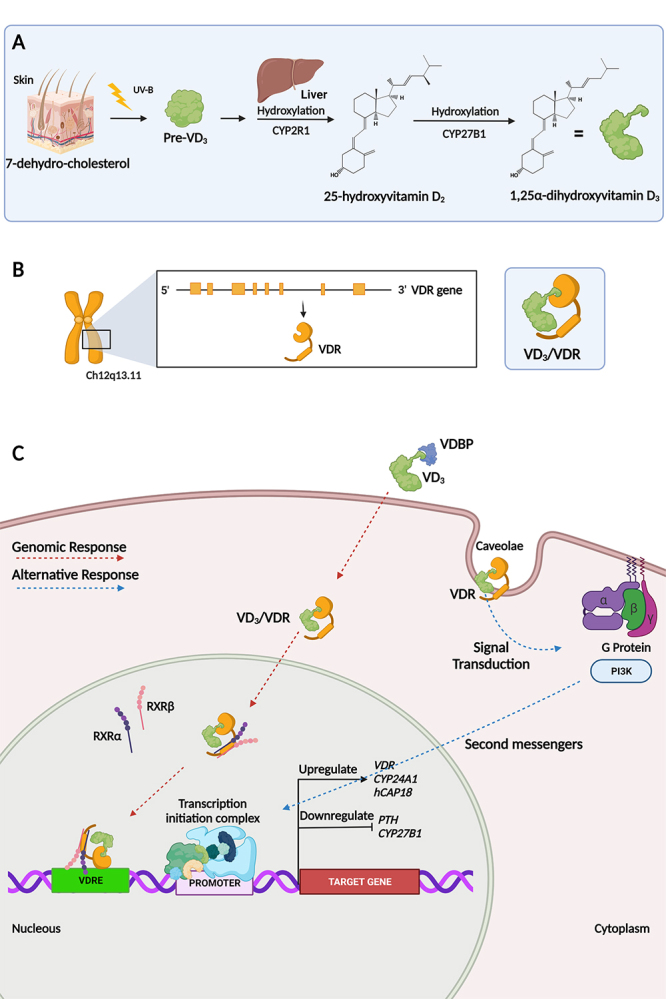
Metabolic and genetic aspects of VD_3_. A) Metabolism of VD_3_. In epidermal cells is found the molecule 7-dehydrocholesterol, which is broken down by UVB radiation from the sun, forming pre-VD_3_. This undergoes first hydroxylation process in liver through CYP2R1 enzyme, having as a product of the reaction to 25-hydroxyvitamin D_2_. This is converted into 1,25α-dihydroxyvitamin D_3_ through the second hydroxylation process through CYP27B1 enzyme. B) Chromosomal localization of the VDR gene. VD_3_ performs its functions by connecting to the VDR, forming VD_3_/VDR. C) Transcriptional complex of VD_3_/VDR. Gene regulation by genomic site (red arrow) occurs when VD_3_ binds to VDBP transporter. The VD_3_/VDR interaction is recruited to nucleus that heterodimerizes with RXRα and RXRβ. These act as transcription factors and bind to the VDREs present in the promoter region of target genes. Gene regulation by means of the alternative site (blue arrow) is a rapid response that occurs when VDR interacts with caveolae and through signal transduction activates the PI3K enzyme that induces intracellular conduction through secondary messages in the promoter region of target genes.

Ultraviolet B (UVB) radiation (280-315 nm), originated from the sun, activates a chemical reaction in the plasmatic membrane of epidermal keratinocytes and dermal fibroblasts in the skin leading to photolysis of B ring from 7-dehydrocholesterol, resulting as product pre-vitamin D_3_, which undergoes a spontaneous, but non-catalytic, thermosensitive process of isomerization (Bikle, 2014). The pre-vitamin D_3_ undergoes a first hydroxylation process on carbon 25 by the enzyme 25-hydroxylase/cytochrome oxidase P450 (CYP2R1), expressed by hepatocytes and stored in the endoplasmic reticulum of these cells (Figure 1A). This first hydroxylation results in VD_2_, the blood stream circulating form with a half-life time of 2 to 3 weeks. VD_2_ is released into the plasma, where binds to a carrier called VD-binding protein (VDBP) and is transported to various tissues in the body (Cheng *et al*., 2004). Another source of VD_2_ is the diet rich in foods such as fatty fish, egg yolks, shiitake mushrooms and liver (Lehmann *et al*., 2015). 

VD_2_ deficiency is considered pandemic, with high prevalence in all population groups, not just risk groups (young children, pregnant women, elderly and negroid population). About one billion individuals have a deficiency in serum levels of this hormone (van Schoor and Lips, 2011; Palacios and Gonzalez, 2014). Ideal VD_2_ serum levels are considered when greater than 32 ng/mL (80 nmol/L). Low serum concentration makes it impossible the functions performed by this hormone and increases the risk of developing several diseases such as hypertension, diabetes, atherosclerosis, TB, and cancer (Heaney, 2008).

VD_2_ is converted into VD_3_ by hydroxylation, an active state with a time of half-life of 4 to 6 hours. The second hydroxylation process, on carbon 1 from A ring, is performed by the enzyme 1α-hydroxylase, a product of the *CYP27A1* gene that is expressed in greater amounts in the cells of the proximal tubules of the kidneys but can also be expressed by other cell types such as leukocytes, especially macrophages (Adams and Hewison, 2010) (Figure 1A). 

VD_2_ performs its functions in the body through two main mechanisms: an endocrine mechanism that allows the regulation of calcium absorption; and an autocrine, which promotes the expression of several genes (van Schoor and Lips, 2011) (Figure 1C). VD_3_ plays many roles in the body, including modulating cell growth, neuromuscular and immune function and reducing inflammation (Norman, 2008; Kamen and Tangpricha, 2010; Boucher, 2012). Many genes that encode proteins that regulate cell proliferation, differentiation and apoptosis are modulated in part by VD_3_. The VDR is expressed in several cell types and the CYP271A enzyme is responsible for local conversion of VD_2_ into VD_3_ (National Institutes of Health, 2020) (Figure 1).

VDR is a member of the steroid hormone receptor superfamily, which includes receptors from retinoic acid, thyroid hormone, sex hormones and adrenal steroids (Margolis and Christakos, 2010; Wang *et al*., 2012). The mechanism of action of VD_3_ is mediated by the VDR, which acts as a transcription factor in target cells after forming a heterodimer with the α and β retinoic acid receptors (RXRα and RXRβ). Once dimerized, the complex binds to the VD response element (VDRE), located in the promoter regions of target genes or at enhancers, to up- or down-regulate their expression (Gil *et al*., 2018) (Figure 1B,C). As the VDR has been found in virtually all cell types, it may explain its multiple actions in different tissues (Wang *et al.*, 2012).

The *VDR* in humans is located on chromosome 12 (12q13.11) and is composed of eight coding exons (2-9) in addition to six non-coding exons (1a-1f), which have alternative splicing, in eight introns. The gene also has two promoter regions (Zella *et al*., 2006) and a tissue-specific promoter is suggested (Zella *et al.*, 2010) (Figure 1).

The VDR protein in humans contains 427 amino acids (Pike and Meyer, 2010; Haussler *et al*., 2013). The two main VDR functional domains are the highly conserved NH2-terminal DNA-binding domain (DBD) and the more variable COOH-terminal ligand-binding domain (LBD). DBD is a cysteine-rich zinc finger region. There are two zinc fingers, each of which contains a single zinc atom in a tetrahedral arrangement with four invariant cysteine ​​residues (Haussler *et al.*, 2013). The LBD is composed of at least 12 α helices [H1-H12; the ligand-dependent activation function (AF2) corresponds to H12] and 3 β sheets (S1-3). VD_3_ binding induces a conformational change that facilitates interaction with the RXR and co-regulatory complexes necessary for transcription of target genes (Christakos *et al*., 2016). 

Several genes can be directly upregulated such as *CYP24A1* or downregulated such as parathyroid hormone (*PTH*) and *CYP27B1*, through VDR activation (Christakos *et al*., 2016). The VD_3_/VDR/RXR complex interacts with basal transcription factors such as transcription factor IIB (TFIIB) and several factors associated with TATAbox binding protein in DNA. VDR-mediated transcription is facilitated by the mediator, a multiprotein complex that functions through the recruitment of RNA polymerase II and promotes the formation of the transcriptional pre-initiation complex (Yin and Wang, 2014) (Figure 1C).

In addition to VD_3_, the VDR-RXR dimer can associate with other molecules such as the p160 family of steroid receptor coactivators 1, 2 and 3, which have histone acetylase (HAT) activity, and are primary coactivators that bind for the AF2 domain of the VDR complex (Christakos *et al*., 2016). Members of the p160 family recruit proteins as secondary coactivators, such as CBP/p300, that also have HAT activity, resulting in a complex of multiple subunits that modify chromatin and destabilise the histone/DNA interaction (Pike *et al*., 2016). 

The result of VDR genomic interactions is the regulation of transcription of multiple genes, in many cases far from the VDR *cis* binding site. However, in some cases, the VDR can still exert a regulatory action in the absence of the VD_3_ (Gil *et al*., 2018). Genetic alterations in the *VDR* can lead to important defects in gene activation, affecting calcium metabolism, cell proliferation and immune function, which can be explained by changes in protein conformation (Jones *et al*., 1998; DeLuca, 2004; Vasilovici *et al*., 2018). The gene comprises more than 60 single nucleotide polymorphisms (SNPs) identified in the coding region. Despite this large number, few are considered functional, and the most studied *VDR* polymorphisms are: *FokI, Cdx-2, TaqI, BsmI and ApaI* (Table 1) (Ozaki *et al*., 2000; Huang *et al*., 2002; Halsall *et al*., 2004; Ralston and Rossant, 2008; Gapska et al. 2009; Savory *et al*., 2009; Luo *et al*., 2011; Luo *et al.*, 2012; Carvalho *et al*., 2015; Salimi *et al*., 2019).

**Table 1 -:** VDR SNPs.

VDR SNP	Function
rs2228570 (*FokI*)	Location: exon 2; Change: C > T. Generates a non-synonymous polymorphism resulting in a change of threonine to methionine. The presence of the restriction site *FokI* C allele, generates a new start codon (ATG) 9 bp after the common starting site, which translates to a shorter truncated VDR protein of 424 amino acids with more transactivation capacity as a transcription factor than the wild type full-length VDR A isoform (VDRA) with 427 amino acids (Luo *et al*., 2011; Carvalho *et al*., 2015; Salimi *et al*., 2019).
rs11568820 (*Cdx2*)	Location: *VDR* promoter region; Change: A > G. Potentiates the binding strength between VDR and its transcriptional complex (Ralston and Rossant, 2008; Savory *et al*., 2009).
rs7975232 (*ApaI*)	Location: intron 8; Change: A > C. Does not change the amino acid sequence of the VDR protein, therefore could affect mRNA stability and the gene expression of *VDR* (Salimi *et al*., 2019).
rs1544410 (*BsmI*)	Location: intron 8; Change: A > G. Could affect mRNA stability and *VDR* gene expression. Could generate an alteration in the splice sites for mRNA transcription or a change in the intron regulatory elements of *VDR* (Ozaki *et al*., 2000; Huang *et al*., 2002; Luo *et al*., 2012).
rs731236 (*TaqI*)	Location: exon 9; Change: C > T. Generates a synonymous change of the isoleucine amino acid in the coding sequence, therefore it does not change the encoded protein, but it could influence the stability of the mRNA (Carvalho *et al*., 2015; Salimi *et al*., 2019).

*Vitamin D and inflammation*

In the last years an immune response role has been described for VD_3_. Its immunomodulatory actions influence innate and adaptive immune responses either by regulating the expression/release of cytokines or by regulating cytokines and cell signalling pathways (Laird *et al*., 2020). The VD_3_/VDR complex can control more than 200 genes involved in cell proliferation, differentiation, apoptosis, and angiogenesis (DeLuca, 2004; Nagpal *et al*., 2005; Holick, 2007).

**Figure 2 -:**
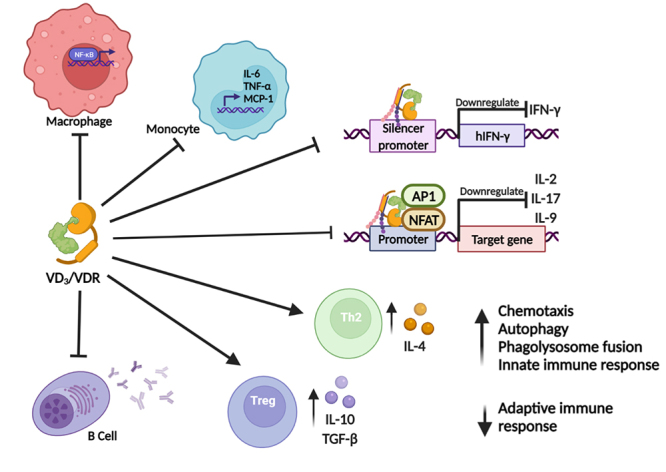
Genetic effect of VDR on different cells of the immune system. In macrophages VDR inhibits activation of NF-kB. In monocytes, the production of IL-6, TNF-α and MCP-1 is inhibited. The VD_3_/VDR/RXR complex promotes *IFN-γ* gene silencing. Blockade of AP-1 and NFAT formation by VD_3_/VDR/RXR complex promotes suppressive effect on transcription of IL-2, IL-9 and IL-17. The immunomodulatory action of VD_3_ inhibits the immune response of Th1 cells and modulates the activities of Th2 and Treg cells, through the production of IL-4, and IL-10 and TGF-β, respectively. VD_3_ inhibits the production of antibodies.

The involvement of VD_3_ in the immune system becomes even more evident when several studies *in vivo* and *in vitro* showed that immune cells, such as DCs, macrophages, B and T cells express the VDR (Yin and Agrawal, 2014) (Figure 2).VD_3_ has been closely associated with modulation of the immune response against different pathogens and acting in the regulation of the adaptive immune response in chronic inflammatory disorders and autoimmune diseases (White, 2008; Tiosano *et al*., 2013; de Albuquerque Borborema *et al*., 2021). It is known that in monocytes, VD_3_ acts by inhibiting the production of IL-6 and TNF and reduces the expression of monocyte chemoattractant protein-1 (MCP-1) which inhibits the activation of NF-κB in macrophages (Sanchez-Niño *et al*., 2012; Zhang et al. 2012; Filgueiras *et al*., 2020) (Figure 2). In general, VD_3_ works by enhancing the innate immune response and inhibiting the adaptive response (Topilski *et al*., 2004). 

The immunosuppressive effect of VD_3_ is correlated with a decrease in the expression and consequent release of inflammatory cytokines, including IL-2 and IFN-γ (Lemire *et al*., 1985).

The repressive effect of VD_3_ on IFN-γ transcription is due to the direct binding of the VD_3_/VDR/RXR complex to a promoter silencer region of its gene (*hIFN-γ*) (Christakos *et al*., 2016). VD_3_ also stimulates the production of anti-inflammatory cytokine IL-4 by Th2 cells (Mahon *et al*., 2003). This hormone has also been reported to play a role in increasing Treg cells, a subset of CD4+ T cells important for inhibiting inflammation and for inducing forkhead box P3 (FOXP3), a transcription factor lineage-specific involved in Treg cell development and function (Jeffery *et al*., 2009; Unger *et al*., 2009; Van Belle *et al*., 2014) (Figure 2)**.**

The stimulatory effects of VD_3_ on the expression of IL-4 and IL-10 and perhaps other cytokines may be indirect, and the VD_3_ immune response may be dependent on the interaction of various cell types and activation states (Christakos *et al*., 2016).

One of the main targets of VD_3_ are DCs. Exposure of differentiated *in vitro* DCs to VD_3_ interferes with their maturation, locking the cells into a semi-mature state. Altered DCs have a reduced expression of MHC class II, costimulatory molecules (CD40, CD80, CD86) and an altered IL12/IL10 ratio (Ferreira *et al*., 2011).

DCs are able to alter the behaviour of T lymphocytes, inducing T cell anergy and increasing levels of apoptosis, while shifting T cell cytokine responses from pro-inflammatory, Th1 and Th17, to a more tolerant profile with Th2 and Treg cells. VD_3_ affects the phenotype and behaviour of DCs through their early and transcription-mediated reprogramming of metabolic pathways, *i.e.,* increased glycolysis and oxidative phosphorylation at the same time (Ferreira *et al*., 2011).

VD_3_ increases the production of β2 defensin and CAMP by macrophages and monocyte-derived keratinocytes increasing their antimicrobial activity (Gombart *et al*., 2005; Dai *et al*., 2010). In addition, this hormone enhances chemotaxis, autophagy, and phagolysosome fusion of innate immune cells (White, 2010). 

VD_3_/VDR inhibits the expression of cytokines by APCs such as IL-1, IL-6, IL-12 and TNF and decreases the expression of a set of cell surface proteins of the MHC in macrophages and development of Th1 and Th17 pro-inflammatory cells, while inducing Treg and Th2 cells, which in turn down-regulate Th1 activity. Thus, this hormone inhibits the production of IL-12 and stimulates the production of IL-10, while down-regulates the expression of some costimulatory molecules, *e.g*., CD40, CD80 and CD86 differentiation clusters, necessary for DCs activation and other APCs, leading to Th1 inhibition. Furthermore, VD_3_ acts directly on T cells by inhibiting the secretion of IL-2, a cytokine essential for the clonal expansion of lymphocytes, and IFN-γ (Christakos *et al*., 2016; Gil *et al*., 2018). 

VD_3_ also inhibits B cell differentiation and antibody production. In addition, inhibits enterocyte apoptosis and promotes the synthesis of antimicrobial peptides and reduces the proliferation of keratinocytes in psoriasis, favouring cell differentiation in both cases (Christakos *et al*., 2016; Gil *et al*., 2018). The VD_3_/VDR complex activates the expression of antimicrobial peptides (AMPs), such as cathelicidins and beta defensins that attack pathogens (Wang *et al*., 2004; Lai and Gallo, 2009). 

VD_3_ suppresses adaptive immunity (Chun *et al*., 2014; Wei and Christakos, 2015). In experimental models, this hormone negatively regulates immune responses mediated by Th1 cells, thus inhibiting the production of pro-inflammatory cytokines, such as IFN-γ, IL-6, IL-2 and TNF (Carvalho *et al*., 2017; Xie *et al*., 2017). Although experimental studies *in vitro* and in animals have produced encouraging results on the immunomodulatory effect of VD_3_, the same cannot be said about studies in humans, since they are discrepant regarding the confirmation of the suppressive effect of this hormone on Th1 cells and on the production of inflammatory cytokines in different diseases (Bendix-Struve *et al*., 2010; Stubbs *et al*., 2010; Seibert *et al*., 2013; Drozdenko *et al*., 2014; Buondonno *et al*., 2017; Zhou *et al*., 2018).

The overall effect of VD_3_ on Th cell differentiation may be mediated by its effect on DCs, which are responsible for T cell differentiation into an effector cell with pro- or anti-inflammatory properties, therefore, modulation of APCs is crucial to initiate and maintain adaptive immune response and self-tolerance (Hu and Wan, 2011). *In vitro* differentiation of DCs in the presence of VD_3_ induces a “tolerance state” characterised by low levels of inflammatory cytokines such as IL-12 and TNF, and increased levels of anti-inflammatory cytokines such as IL-10. These cells further induce Treg cell differentiation and autoreactive T cell apoptosis (Penna and Adorini, 2000; Piemonti *et al*., 2000; van Halteren *et al*., 2004; Unger *et al*., 2009).

More studies are needed to completely elucidate the mechanisms of VD_3_ in inflammation and immune response.

*Vitamin D and tuberculosis*

Exposure to sunlight has been known for over centuries to help in the treatment of TB, although the first indicator of VD with antimicrobial activity against *Mtb* was from studies only in the 1980s, in which VD stimulation of monocytes and macrophages infected with the bacillus showed reduced mycobacterial burden (Crowle *et al*., 1987; Cohen *et al*., 1992; Liu *et al*., 2007; Holick and Chen, 2008).

**Figure 3 -:**
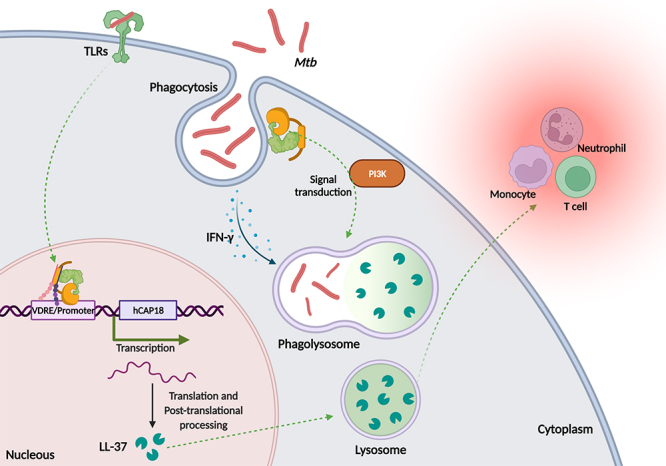
Mechanism of action of the *Mtb* in front of VD_3_. The *Mtb* is phagocytized by the alveolar macrophages, is internalized in phagosome, fuses with lysosome and forms the phagolysosome via IFN-γ. Bacilli remain in the cytoplasmic vacuole and recruit immune cells to maintain the innate response. *Mtb* can be recognized through TLRs and induces several cascades of intracellular signaling. The presence of VD_3_ affects *Mtb* infection through two mechanisms: recruitment of the VD_3_/VDR/RXR complex to the VDRE present in promoter region of the *hCAP-18* gene and consequent production of LL-37, antimicrobial peptide, and formation of IFN-γ independent phagolysosome with activation of PI3K signal transduction.

People with active TB often have VD_2_ deficiency (Desai *et al*., 2012). VD_3_ can affect *Mtb* infection by several immunological defence mechanisms, among them is the production of the specific antimicrobial peptide for *Mtb*, LL-37, through the activation of the expression of hCAP-18 gene, precursor of this cathelecidin (Selvaraj, 2011; Dini and Bianchi, 2012). Antimicrobial peptides such as defensins and cathelicidins are involved as a first line of defence in preventing infections, including TB (Rivas-Santiago *et al*., 2008) (Figure 3).

Although cathelicidin are widely distributed in mammals, LL-37 is the only member of the cathelicidin family that has been identified in humans, being found in alveolar macrophages, lymphocytes, neutrophils, and epithelial cells (Martineau *et al*., 2007; Rivas-Santiago *et al*., 2008). In addition to direct bactericidal activity, LL-37 also modulates the immune response by attracting monocytes, T cells, and neutrophils to the site of infection (Martineau *et al.*, 2007). The dose-dependently presence of VD_3_ in neutrophils and macrophages upregulates the *hCAP-18* by binding the VD_3_/VDR/RXR transcriptional complex to the VDRE present in the promoter region of this gene (Liu *et al*., 2007) (Figure 3). Increased activation of TLRs by the VD_3_ also results in the production of defensin-2 and cathelecidins (Adams *et al*., 2009).

VD_3_ reduces *Mtb* viability by increasing phagosome and lysosome fusion in infected macrophages (Chocano-Bedoya and Ronnenberg, 2009). The pathways used to promote VD-induced phagolysosome formation are independent of classical IFN-γ-dependent macrophage activation and involve products of phosphatidylinositol-3-kinases (PI3K), which helps to regulate the transport of endosomes into lysosomes (Liu *et al*., 2007) (Figure 3). Furthermore, the presence of the VD_3_ is essential for the IFN-γ-mediated antimicrobial function of macrophages (Fabri *et al*., 2011). Another relevant role of VD_3_ in TB control may be its modulating effect on the T cell phenotype, balancing Th1 and Th2 responses (Lin and Flynn, 2010).

Exposure of human monocytes to *Mtb* upregulates the expression of *CYP27B1* and the *VDR* itself, thus increasing the cell’s ability to produce VD_3_ at the site of infection and to respond to this hormone more efficiently. However, monocytes differentiate into different macrophage profiles that play different roles in the immune response (Sassi *et al*., 2018). For example, macrophages formed after IL-15 stimulation respond to VD_3_ stimulation by increasing their antimicrobial activity, while phagocytic macrophages obtained after IL-10 stimulation are weakly influenced by VD_3_ levels, regardless of their high phagocytic activity (Kim *et al*., 2018).

VD_3_ increases the defence capacity of macrophages by inducing their differentiation, phagocytic capacity, and antimicrobial activity, also increasing the expression of cathelicidin. Furthermore, VD_3_ inhibits monocyte proliferation and promotes the differentiation of monocytes into macrophages, these effects are mediated by cell surface Fc receptors upregulation and by increase of cellular respiration. VD_3_ inhibits the proliferation and maturation of DCs, as well as its immune stimulating properties, leading to the induction of Treg cells. Consequently, VD_2_ deficiency results in a state less tolerant to foreign antigens (Christakos et al. 2016).

Genetic alterations in *VDR* can lead to defects in gene activation or changes in VDR protein structure, both of which can affect VD_3_ cell functions. Several polymorphisms in the *VDR* may also be linked to each other or to unidentified genes that are important determinants of the risk of developing active TB (Lee *et al*., 2016; de Albuquerque Borborema *et al*., 2020).

*Vitamin D and COVID-19*

Recently, a few studies have demonstrated a potential link between the deficiency of VD_3_ and several diseases (Dankers *et al*., 2017; Mamani *et al*., 2017; Bouillon *et al*., 2019; Pagano *et al*., 2020; Sulli *et al*., 2021). Some authors hypothesised that VD_3_ insufficiency may compromise respiratory immune function, increasing the risk of COVID-19 severity and mortality (Daneshkhah *et al*., 2020; Darling *et al*., 2020; Raharusun *et al*., 2020; Grant *et al*., 2020; Hastie *et al*., 2020; Ilie *et al*., 2020; Watkins, 2020). It seems that, at sufficient levels, VD_3_ controls the release of antiviral peptides, able to directly prevent viral replication (Gombart *et al*., 2005; Wang *et al*., 2010; Greiller and Martineau, 2015).

In view of its immunomodulatory action, VD_3_ uses three different mechanisms to reduce the risk of several viral infection tract respiratory, such as Influenza A, respiratory syncytial virus (RSV) and rotavirus, which are: physical barrier - mucus secretion, in which presence of fatty acids and enzymes eliminate the pathogen -, innate cellular immunity and adaptive immunity (Greiller and Martineau, 2015). 

In innate cellular immunity, there will be greater production of anti-inflammatory cytokines, due to the reduction of pro-inflammatory cytokines and consequent decrease in the activation of cytokine storm (Cantorna *et al*., 2015). A study realised in an animal model with bleomycin-induced pulmonary fibrosis and human lung fibroblast cell line (HLFCLs) shows that VD_3_ exerted activity in lung tissue and protective effects in interstitial pneumonia, because in HPFCs there was an increase in *VDR* mRNA levels and genes that metabolize VD_3_, and in addition, there was a suppression in the dosage of pro-inflammatory cytokines. In the animal model, there was a significant improvement in the symptoms of fibrosis, and the expression of genetic markers was reduced on a diet rich in VD_3_ (Tsujino *et al*., 2019).

*In vitro* studies demonstrate that this hormone plays a significant role in local “respiratory homeostasis” in this case, the VD_3_/VDR/RXR complex, described in a previous topic, binds to the promoter region of antimicrobial peptides, cathelicidin and defensins, which has direct activity against a spectrum of pathogens, including gram-positive and gram-negative bacteria, enveloped and non-enveloped viruses, and fungi, with an increase in its expression and neutralization of endotoxins, and consequently the reduction of the viral replication rate, which promotes chemotaxis of immune cells to the inflamed organs and induces autophagy (Bikle, 2016; Zdrenghea *et al*., 2017). 

Another study evaluated the effect of the treatment of human cathelicidin LL-37 in mice infected with the Influenza A virus. In this case, infected mice had decreased levels of pro-inflammatory cytokines in the lung compared to untreated mice, suggesting that cathelicidins may be key components of the immune system and in reducing infection and inflammation, as well as therapy derived from this peptide may provide protection (Barlow *et al*., 2011).

A recent *in silico* study reported the possible involvement of TLRs that respond to viral ligands, such as TLR3, TLR7 and TLR8, and specifically TLR4 that responds to bacterial ligand, in the recognition and induction of inflammatory response to *SARS-CoV-2*, in which a hydrophobic and hydrophilic interaction between TLRs with spike protein (TLR-spike) was observed (Choudhury and Mukherjee, 2020). In contrast, a preliminary study reveals that oral administration of cathelicidin improved the symptoms of 11 patients with mild COVID-19, suggesting that improvement in circulating levels of VD_3_ may be a constituent in the initial host defence against *SARS-CoV-2* infection through the production of antimicrobial peptides (Wan S *et al*., 2020). 

**Figure 4 -:**
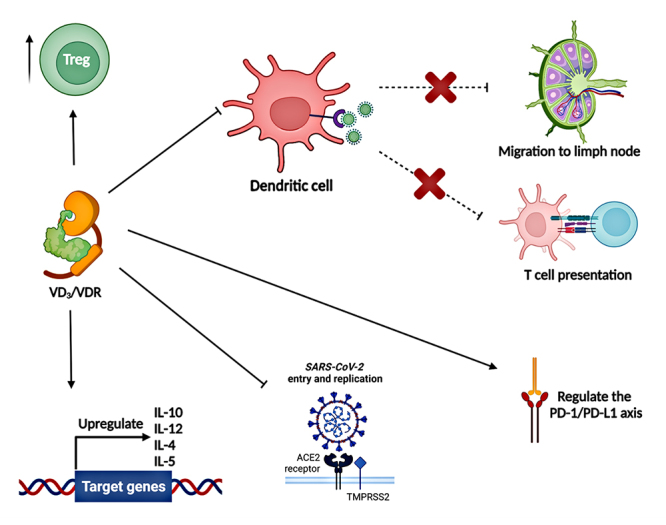
Immunogenetic activity of VD_3_ in COVID-19. VD_3_ induces the production of Treg cells. The VD_3_/VDR complex induces expression of anti-inflammatory cytokines. VD_3_ inhibits the differentiation and maturation of DCs, preventing migration to lymph nodes and activation of T cells of pro-inflammatory profile. VD_3_ regulates expression of PD-L1. The increase in the activity of the alternative axis of RAS induces greater expression of ACE2, VD_3_ may decrease viral replication.

In adaptive immunity, T and B cells present important activities in the immune response to a viral infection and VD_3_ can act to contain the pathogen. Individuals with COVID-19 seem to present functional exhaustion of T and B cells, because the main manifestation of severe *SARS-CoV-2* infection is lymphopenia (Qin *et al*., 2020; Bae *et al*., 2022). However, VD_3_ may contain this process through immunomodulation on the adaptive immune response (Bikle, 2022). In severe COVID-19, DCs are activated when they find the *SARS-CoV-2* and migrate to the lymph nodes, however, VD_3_ inhibits DCs its differentiation and maturation, as well the expression of costimulatory molecules, such as CD40, CD80 and CD86, these aspects induce DCs to present a tolerogenic phenotype, reducing antigen presentation and T cells activation (Vanherwegen *et al*., 2017; Bikle, 2022). Still, to modulate the immune system, this hormone can induce the production of Treg cells, induce gene transcription of anti-inflammatory cytokines such as IL-10, and a phenotypic change from Th1 to Th2 (Kumar *et al*., 2021) (Figure 4).

To better understand how the action of the adaptive immune response occurs against *SARS-CoV-2* and VD_3_ performance in this context, it is interesting to mention about the immune checkpoint inhibitory molecules expressed on the surfaces of T cells, B cells, DCs and natural killers (NK), such as programmed death-1 (PD-1) and their programmed death-1 linker (PD-L1), which play a crucial role in severe inflammation (Wan S *et al*., 2020). 

PD-1, when expressed, is a potent immune regulator because it indicates an activation threshold before an immune response is initiated, thus limiting the activity of T and B cells preventing the proliferation and differentiation of T cells (Schönrich and Raftery, 2019; Aygun, 2022).

In a clinical and experimental study, a positive regulation of *PD-L1* mRNA levels in individuals with COVID-19 was reported when compared to controls. The results showed positive regulation of *PD-L1* in epithelial cells infected with *SARS-CoV-2* and negative regulation of this gene in immune cells such as monocytes, neutrophils and CD4+ T cells, suggesting that this gene may present a positive prognosis to individuals with COVID-19 (Sabbatino *et al*., 2021).

Interestingly, PD-1/PD-L1, is regulated through several signalling pathways activated by pro-inflammatory cytokines (Aygun, 2022). As already reported, VD_3_ has a potential role to reduce inflammation and a recent study reported that this hormone can regulate PD-1/PD-L1 axis by increasing PD-L1 when they are too low or decreasing PD-L1 when are too high, *i.e.,* through T-cell production. This suggests that sufficient levels of VD_3_ decrease pro-inflammatory cytokine levels and consequently obtain a favourable immune response to the individual by regulating this pathway (Morita *et al*., 2021).

In addition to all the aspects mentioned above on VD_3_, it is also known that the deficiency of this hormone may promote dysregulation of the renal angiotensin-aldosterone system (RAAS), which may predispose the individual to a higher risk of cardiovascular diseases and lung diseases, risk features to severe COVID-19 (Bae *et al*., 2022). The RAAS system operates through two mechanisms: classic and alternative. The classical mechanism is composed of angiotensin converting enzyme (ACE), angiotensin-II (Ang-II) and AT1R, called Ang-II/ACE/AT1R axis, triggering vasoconstriction and production of reactive oxygen species (ROS). The alternative, or counter-regulation, consists of angiotensin-converting enzyme 2 (ACE2), angiotensin 1-7 (Ang1-7) and Mas receptor (MasR), called the Ang1-7/ACE2/MasR axis, which has a vasodilator role and inhibits ROS production. This system is a homeostatic regulation pathway, and the balance between ACE/ACE2 is essential for electrolyte and tissue balance (Patel *et al*., 2017; Paz Ocaranza *et al*., 2020).

Thus, when RAAS dysregulation occurs, the classical axis presents excessive activity when compared to the alternative axis. Generally, individuals with systemic arterial hypertension, heart failure or other comorbidity use medications such as ACE inhibitors (iACE) and angiotensin receptor blockers (ARBs), which promote higher ACE2 production (de Kloet *et al*., 2010; Babajani *et al*., 2021). ACE is the gateway to *SARS-CoV-2* through its S protein, competing with Ang-II blocking ACE2 activity, promoting vasoconstriction and endothelial dysfunction. Therefore, pre-existing comorbidities are related to developing a higher risk of severe COVID-19 and higher mortality (de Lucena *et al*., 2020; Beyerstedt *et al*., 2021).

VD_3_ acts inhibiting the expression of ACE and stimulating the alternative axis activity, while negatively regulating the classical axis (Babajani *et al*., 2021). Even if there is greater expression of ACE2, this hormone reduces the entry and replication of viruses, including *SARS-CoV-2*, through previously mentioned mechanisms.

In fact, the specific action of VD against *SARS-CoV-2* is not yet clearly understood and further genetic studies are still needed to prove the beneficial performance of this hormone in COVID-19 prognosis, but the literature cited so far demonstrate possible mechanisms that are similar to its antimicrobial and antiviral activities.

### 17β-estradiol (E_2_)

Estrogen refers to a group of female mono phenolic steroid hormones that performs numerous activities throughout the body. Its chemical structure consists of 17 carbon-carbon bonds arranged in four molten rings (Fuentes and Silveyra, 2019). The four estrogens are denominated C_18_ steroids since it contains 18 carbons (Patel *et al*., 2018). They consist of a benzene ring, phenolic group, and a ketone group (estrone or E_1_) or one group (17β-estradiol or E_2_), two groups (Estriol or E_3_) or three hydroxyl groups (Estretrol or E_4_) (Fuentes and Silveyra, 2019). E_2_ is the estrogen with the highest biological activity and consists in a phenolic ring, a hydroxyl (OH) at position 17 and a grouping in the β conformation (Jia *et al*., 2015).

In women at puberty and pre-menopausal periods, estrogen is mainly synthesized in the ovaries, but can also be produced in the liver and adipose tissue, using cholesterol as a precursor, and regulating various physiological processes such as growth, reproduction, development, and cellular differentiation (Fan *et al*., 2019; Fuentes and Silveyra, 2019). In post-menopausal women and men, the synthesis of E_2_ occurs through the conversion of the male sex hormone, testosterone, and androstenedione, from the estrone under the action of the aromatase-converting enzyme of cytochrome P450 (Österlund and Hurd, 2001; Jia *et al*., 2015; Saczko *et al*., 2017). 

**Figure 5 -:**
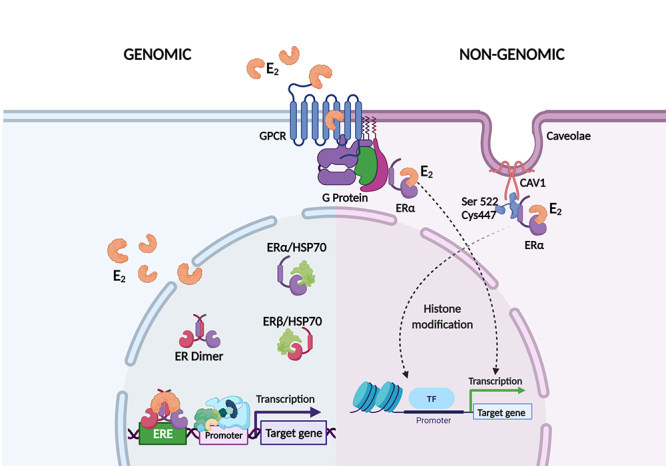
Genomic and non-genomic mechanisms of E_2_. In genomic signaling, E_2_ binds to ERα and ERβ receptors in the cytoplasm, which undergo a conformational change, dimerize, and are translocated to nucleus. ERα e ERβ act as transcription factors, and E_2_/ER complex binds to ERE sequences near promoter region inducing transcription. In non-genomic signaling, plasma membrane receptors activate PI3K/AKT. The ERα subunit binds to Cys447 and also binds to caveolae through Ser522, inducing transcriptional activity due to histone modification. E_2_ also has a link with GPCRs that interacts with E_2_/ER complex and induces transcriptional activation.

The physiological functions of E_2_ are regulated by binding at estrogen receptors (ERs), the subtypes ERα and ERβ, which perform long-term or rapid actions, *i.e.,* acting through classical or non-classical receptors, genomics (nuclear) or non-genomic (extranuclear), respectively, in which it controls mechanisms of gene expression, protein modification and cell signalling (Moulton, 2018; Trenti *et al*., 2018) (Figure 5). ERα are found mainly in reproductive tissues (uterus, ovary), breast, adipose tissue, and liver, while Erβ is found in the ovary, cardiorespiratory system and immune system (Jia *et al*., 2015).

The classical or genomic mechanisms are mediated by ERα and ERβ isoforms with 56% homology and ensembles homo or heterodimers (α-α, β-β, α-β) before binding to estrogen response elements (EREs) to alter gene expression (Moulton, 2018). The genes encoding ERα and ERβ are *ESR1* and *ESR2*, respectively. *ESR1* is located on chromosome 6q25.1 and codes a protein with 595 amino acids while *ESR2* is located at 14q23.2 and ERβ present 530 amino acids (Cohen *et al*., 1992; van Halteren *et al*., 2004). Both ERs have a DNA binding domain and six regions, denominated A to F and divided into four functional domains: amino-terminal domain (domains A and B or NTD) with ligand function domain 1 (AF-1); a DNA binding domain (C domain or DBD) (van Halteren *et al.*, 2004), which contributes to the dimerization of the ER and to the binding to EREs specific sequences and an activation to ligand 2 (AF-2) domain; a D domain which is a region that contains the nuclear localization signal and when binding to estrogen allows the receptor-ligating complexes to perform translocation to the nucleus (Jia *et al*., 2015; Saczko *et al*., 2017); and the E/F domain with the carboxy terminal contains the estrogen connection area together with other co-activators (Fuentes and Silveyra, 2019) (Figure 5).

Thus, genomic signalling occurs as follows: the nuclear receptors of estrogen ERα and ERβ act as transcription factors activated by ligands. After E_2_/ERα or ERβ binding in the cytoplasm, a conformational alteration occurs inducing the receptor dimerization (Le Dily and Beato, 2018). This complex is translocated to the nucleus, where it binds to ERE (CAGGTCA) sequence (Trenti *et al*., 2018; Fuentes and Silveyra, 2019) (Figure 5).

The ERs non-genomic mechanism promotes faster responses (in minutes) through receptors in the plasma membrane that are often associated with the activation of protein-kinase cascades such as PI3K with protein kinase B (PKB or AKT), intracellular calcium mobilization, cyclic adenosine monophosphate (cAMP) generation, potassium current modulation, phospholipase C activation, and NO production (Trenti *et al*., 2018). 

The non-classical mechanism, also known as membrane-initiated steroid signalling (MISS), is mediated by a subunit of ERα associated with a palmitoylation site (Cys447 in humans) with the plasma membrane cytosolic portion. In the membrane, the ERα is connected to the caveolae by direct connection to caveolin-1 (CAV-1) through Ser522, performing transcriptional activity once MISS leads to histone modification and chromatin structure (Björnström and Sjöberg, 2005; Pérez-Cremades *et al*., 2018). E_2_ can also bind to G protein associated with receptors with coupled membrane (GPCRs) and trigger the signalling in many cell types such as immune cells. The E_2_/ER complex can to bind to the receptor of estrogen coupled to G receptor protein 1 (GPER1) and lead to several gene transcriptional activation (Fuentes and Silveyra, 2019).

*E*_*2*_ *and inflammation*

ERs are located in several immune system cells able to influence gene regulation (Di Florio *et al*., 2020). Furthermore, E_2_ exerts anti- and pro-inflammatory effects, depending on the cell target, serum levels, organ microenvironment and expression levels from the receptors (Trenti *et al*., 2018; Fan *et al*., 2019). 

In the adaptive immune system, ERs are present in thymocytes and thymus epithelial cells, modulating T cells differentiation and function (Brown and Su, 2019) promoting production of IFN-γ by Th1 cells in humans and mice, via gene promoter direct interaction (Karpuzoglu-Sahin *et al*., 2001). E_2_ also regulates the anti-inflammatory exchange from Th2 by Serine/threonine-protein kinase 1 (SGK1) activation and promotes Treg cells expansion by regulating FOXP3 expression (Polanczyk *et al*., 2005).

In the innate immune system, ERs functions control signalling pathways in DCs and macrophages. E_2_/ERα complex may promote pro-inflammatory cytokines in response to TLR stimulation in DCs and macrophages. Interestingly, E_2_ inhibits neutrophil activation through metabolic oxidation reduction and adhesion to endothelial cells via positive regulation of the anti-inflammatory protein annexin A1. This mechanism attenuates the release of more potent pro-inflammatory cytokines such as TNF, IL-1β and IL-6 (Hughes *et al*., 2013). E_2_/ERα complex seems to fine coordinate a balanced pro-inflammatory response by promoting cell differentiation, MHC-II expression and induces IL-6, IL-23, IL-12 and IL-1β production (Douin-Echinard *et al*., 2008; Kadel and Kovats, 2018). Altogether, those mechanisms act upon tolerogenic phenotype and decreased pro-inflammatory cytokines and chemokines expression by upregulating anti-inflammatory cytokines such as IL-10 and TGF-β (Kadel and Kovats, 2018), *i.e.*, decreasing Th1 and increasing Th2 cytokines. Additionally, E_2_ also reduces PI3K inhibitory signalling and AKT phosphorylation in macrophages, due to the activation of NF-κB impairment (Kovats, 2015).

E_2_ has played a pleiotropic role in pro-inflammatory cytokines synthesis, depending on cell type and dose (Kovats, 2015). At increased levels, E_2_ inhibits pro-inflammatory cytokines such as TNF, IL-1β, IL-6, MCP-1, iNOS, MMPs and activity of NK cells, while also stimulates anti-inflammatory pathways such as IL-4, IL-10, TGF-β, tissue inhibitor of MMP (TIMP), and osteoprotegerin. Meanwhile, at lower concentrations, E_2_ has opposite activity by stimulating TNF, IFN-γ, IL-1β release and NK cells activity (Straub, 2007). 

By preventing the activation of the NF-κB transcription factor family, responsible for the activation of several inflammatory genes, E_2_ assures its anti-inflammatory role. In the presence of inflammatory stimuli such as lipopolysaccharide (LPS) or TNF, E_2_ activates a rapid response through a non-genomic mechanism, involving the interaction between hormone-activated E_2_/ERα and PI3K, which inhibits p65/RelA (member of NF-κB family) nuclear translocation, blocking inflammatory genes transcription without altering inhibitory-κB kinase (IKK)-Iκ-Bα pathway, that is targeted by several NF-κB inhibiting drugs (Ghisletti *et al*., 2005). 

Iκ-Bα is responsible for binding to NF-κB reducing its access to DNA, while IKK, responsible for inflammatory stimuli, phosphorylates Iκ-Bα signalling it for degradation, which then releases NF-κB to nucleus translocation and regulates the expression of targeted gene. In a study using TNF treated rat aortic smooth muscle cells, E_2_ inhibits p65 binding at promoters from inflammatory genes via E_2_/ERβ, while also enhances Iκ-Bα synthesis, therefore augmenting the NF-κB suppression (Xing *et al*., 2012). Also, by activating the intracellular ERα, E_2_ shortens the pro-inflammatory stage in macrophages, leading to an IL-10-dependent “acquired deactivation” phenotype, which results in decreased inflammation and restoration of tissue homeostasis (Villa *et al*., 2015). 

With its broad activity, E_2_ rapidly decreases TLR4 expression in murine macrophages, through G protein-coupled receptor 1 (GPR1) action (Rettew *et al*., 2010). With low TLR4 expression in alveolar macrophages less allergic airway inflammation is noted since inflammasome nucleotide-binding oligomerization domain leucine-rich repeat and pyrin domain containing 3 (NLRP3) is not recruited/activated. In fact, in female C57BL/6 mice with reduced *NLRP3* mRNA levels and downstream products, caspase-1 and IL-1β, resulted in reduced airway inflammation (Cheng *et al*., 2019). 

*Women immunologic profile over the lifetime course*

Susceptibility to infectious diseases between men and women varies not only according to gender but also according to age. In an intrauterine environment, adverse conditions can cause epigenetic adaptations, leading to altered gene activity that can remain throughout life, which includes immune programming (Gluckman *et al*., 2005). Many immunological response differences are observed throughout life, however, puberty and the reproductive senescence are remarkable especially in females, which reinforces the hormones role in immune response modulation (Klein and Flanagan, 2016).

Starting in the intrauterine phase, female fetuses have greater adaptability to intrauterine stress than males, which tend to be more chronically inflamed (Klein and Flanagan, 2016). Comparing female and male fetuses, the first ones present greater adaptability to intrauterine stress and lower placenta inflammation in premature when compared with those from male fetuses. This may provide female survival benefits, with better cardiovascular stability to lower levels of circulating cytokines (Goldenberg *et al*., 2006). Still in intrauterine life, male fetuses have an increased inflammatory response as a reflection of the exacerbated activation of the innate immune response by androgen hormones (Carr *et al*., 1983; Klein and Flanagan, 2016).

At birth, the transition from placental to the external environment and the bombarding with new antigens, present no difference between sex hormones. However, male new-borns have a higher monocytes count compared to females up to 13 months of age when in environments with high pathogenic load (Bellamy *et al*., 2000). Male individuals are believed to continue to develop a more active and efficient innate immunity, compared to females, early in life. This may explain the higher NK cell count in boys when compared to girls at the same age (Lee *et al*., 1996). In addition, a study performed in Nigerian children demonstrated that between 5 and 12 years, females present a lower IgA level, but equivalent levels of IgG and IgM compared to male (Obiandu *et al*., 2013) (Table 2).

**Table 2 -:** Lifetime immune profile.

Age	Differences
*Intrauterine life*	*Woman:* -Increased adaptability to stress (Klein and Flanagan, 2016); -Less inflammation of the placenta; -Lower production of cytokines; -Greater cardiovascular stability. *Man:* - Increased inflammatory response due to exacerbated activation of the innate immune response by androgen hormones (Carr *et al*., 1983; Klein and Flanagan, 2016).
*0-13 months*	*Man:* - High monocyte count when in high pathogenic load environments (Bellamy *et al*., 2000).
5-12 years	*Woman:* -Low levels of immunoglobulin A (IgA), but equivalent levels of IgG and IgM (Klein and Flanagan, 2016; Xiaoxia Lu *et al*., 2020). *Man:* -Increased NK cell count; -Enhanced innate immune response (Lee *et al*., 1996).
*Puberty*	*Woman:* - More active inflammation. - Higher CD4^+^ T cell count; - Higher proportion of CD4^+^/CD8^+^ T cells. *Man:* -Higher number of Treg (Afshan *et al*., 2012).
*Adult life/Childbearing age*	*Woman:* -Higher expression of inflammatory markers; -Less susceptibility to infections (Kovats, 2015; Trenti *et al*., 2018).
*Pregnancy*	- Unique immune system (Mor and Cardenas, 2010); - Immune cells are regulated by E_2_, inhibiting TNF, IL-1β, and IL-6 (Pierdominici *et al.*, 2010).
*Elderly/menopause*	*Woman:* - Suppression of the immune system (Castelo-Branco and Soveral, 2014); - Increased release of pro-inflammatory cytokines (IL-1, IL-6, TNF) and inhibition of IFN-γ release and B and T cells recruitment. *Man:* - Increased expression of IL-6R (Maggio *et al*., 2006); - Decreased activation of the immune system and inflammatory cytokines (Kumru *et al*., 2004).

During puberty, sex steroid hormones play a central role in the immune system, with the female hormonal profile being more intense in the inflammatory aspect (Yang and Kozloski, 2011). Regarding sex, women have a higher CD4+ T cell count and a higher proportion of CD4+/CD8+ T cells, while men have a higher number of Treg cells (Afshan *et al*., 2012). Women at childbearing age have higher levels of inflammatory markers, but slower rates of increased inflammation with increasing age. This may suggest the interaction of sex hormones with inflammatory processes such as vascular disorders and autoimmunity (Tsujino *et al*., 2019). The E_2_ modulating role has been investigated since its hypothetical anti-inflammatory effect that can improve the host’s resistance to degenerative diseases to its pro-inflammatory role that can protect women from infectious diseases (Kovats, 2015; Trenti *et al*., 2018) (Table 2). 

Female reproductive senescence due to depletion of ovarian oocytes and loss of ovarian steroids decreases the levels of sex hormones that can interact with these processes and contribute to age changes in inflammation (Giefing-Kröll *et al*., 2015). As age increases, hormone concentrations go the opposite direction and a faster process of hormone depletion in women is observed, while the immune system of both sexes decreases its efficiency it increases the risk of chronic diseases, especially for women (Castelo-Branco and Soveral, 2014) (Table 2). 

Both innate and adaptive immune responses decrease with age and differ between women and men. After menopause, there is a significant increase in IL-1, IL-6, and TNF and a decrease of IFN-γ serum levels (Deguchi *et al*., 2001). In men, testosterone plays an immunosuppressive role in the production of inflammatory cytokines and its decline with ageing is associated with increased serum levels of IL-6 receptor (IL-6R) (Maggio *et al*., 2006). The humoral and cell-mediated immune responses to antigenic stimulation, vaccination and infection are greater among women than men (Fish, 2008). Women also have higher baseline levels of Ig and higher antibody responses to viruses at any age than men (Klein *et al*., 2010). On the other hand, menopausal women have a reduction in the number of B and T cells and a significant increase in the concentrations of IL-1β, IL-6 and TNF (Kumru *et al*., 2004) (Table 2).

*E*_*2*_ *and tuberculosis*

TB is one of the most studied models of sexual dimorphism in respiratory tract infections. Although the prevalence of TB in men depends on geographic region, there is a general trend for the male/female ratio affecting more males with worse outcomes in developing countries, including a higher risk of mortality (Nhamoyebonde and Leslie, 2014; Nnadi *et al*., 2016; World Health Organization, 2021). This male gender prevalence is also observed when other risk factors such as HIV infection, diabetes mellitus and smoking are combined (Pimpin *et al*., 2011; Khan *et al*., 2015; Cabrera-Gaytán *et al*., 2016). 

Men not vaccinated with Bacille Calmette-Guerin (BCG) have been reported to exhibit a stronger IFN-γ response in the purified protein derivative (PPD) skin test than women, which suggests that males exhibit a stronger immune response that may be associated with an uncontrolled inflammatory process and poor health prognosis during *Mtb* infection (Cabrera-Gaytán *et al*., 2016). During TB infection, there is also a differential immune response characterized by females having higher levels of CXC Motif Chemokine Ligand 9 (CXCL9) while males exhibiting higher levels of the growth factor derived from platelet subunit B (PDGFB), from serum C-reactive protein (CRP) and from specific antibodies against *Mtb*, highlighting a stronger innate and humoral immune response in men (Brown *et al*., 2016; Chavez *et al*., 2016).

In addition, plasma levels of anti-*Mtb* drugs such as isoniazid and pyrazinamide are lower in men than in women. This may be related to a worse outcome in treated men (Ramachandran *et al*., 2017). Interestingly, this higher proportion of affected males is not observed in children, suggesting the role of sex hormones in the pathogenesis of TB (Neyrolles and Quintana-Murci, 2009; Stival *et al*., 2014).

Older women with reduced E_2_ levels (post-menopausal women) have been reported to have an increased risk of *Mycobacterium*-produced chronic lung infections (Chan and Iseman, 2010). Decreased levels of dehydroepiandrosterone (DHEA), an endogenous intermediate in the conversion of cholesterol to estrogens and androgens, have also been reported in elderly women to be associated with infection with *Mycobacterium avium*, a non-tuberculous mycobacterial lung infection (Danley *et al*., 2014). These studies suggest that non-tuberculous mycobacterial infections are also influenced by hormones, which in turn explains the sexual dimorphism in general mycobacterial infections in the respiratory tract (Vázquez-Martínez *et al*., 2018).

Miliary TB or disseminated TB is an uncommon and severe type of extrapulmonary *Mtb* infection. This disease can lead to catastrophic outcomes such as adrenal, hepatic, and pancreatic insufficiency, meningitis, and splenomegaly. With a high mortality rate (15%-20% in children and 25%-30% in adults), its incidence is difficult to record due to high underreporting (Rosenthal *et al*., 2019). On the other hand, patients with genetic susceptibility, immunodeficiency, malnutrition, T2DM, smoking and alcoholism are more susceptible to this clinical manifestation. Among the few case reports in immunocompetent patients, the highest frequency is in healthy adolescents (Hilal *et al*., 2014; Rosenthal *et al.*, 2019). It is proposed that individuals at puberty have a higher risk of disseminated TB due to hormonal changes that would provide an environment of easy colonization and dissemination for *Mtb* (Wilcox and Laufer, 1994).

*In vitro* studies showed that E_2_ is capable of inducing a Th1-mediated pro-inflammatory immune response (Ahmed *et al*., 1999), while testosterone would act by inhibiting the immune response. In an animal study, it was found that castrated mice, which have low levels of androgens and testosterone, had a less harmful clinical outcome than uncastrated mice (Bini *et al*., 2014) which corroborates the potential immunosuppressive effects of androgen hormones in TB. 

It is well established in the literature that women have a more efficient immune system in fighting infections, and that androgen hormones such as E_2_ play an important role in containing the disease. On the other hand, the mechanisms by which this hormone performs these activities and which gene pathways are activated have not yet been established, requiring further studies. 

*E*_*2*_ *and COVID-19*

E_2_ acts as a potential key player in providing immunity against certain viral infections. It is associated with providing immunity against acute lung inflammation and the influenza virus, modulating the cytokine storm, and mediating adaptive immune changes (Shabbir *et al*., 2020). According to WHO, even though men and women have almost equal rates of infection by COVID-19, men tend to have more severe complications, higher rates of hospitalization and deaths (Global Health 50/50, 2022; World Health Organization, 2022).

For most infectious diseases, women are consistently observed to create a more effective immune response than men (Robinson *et al*., 2011). In general, the female immune system responds more efficiently to pathogens, producing greater amounts of IFN and antibodies. However, this protective effect, mediated mainly by E_2_, is attenuated in post-menopausal women (Straub, 2007). For coronaviruses in particular, women have demonstrated a consistent survival advantage over men both in the current COVID-19 pandemic, as well as the 2003 *SARS-CoV* and 2012 *MERS-CoV* epidemics, with substantially lower-case fatality rates (Karlberg, 2004).

Sex differences in inflammation have been well documented and attributed to several factors. Although most immune regulatory genes are encoded by the X chromosome, resulting in a generally stronger immune response in women, this sex difference in inflammatory response is postulated to be largely driven by sex hormones (Giefing-Kröll *et al*., 2015). Although E_2_ plays a complex role in immune system modulation, often in a dose-dependent manner, it is reported to have an anti-inflammatory effect at normal physiological levels in pre-menopausal women (Straub, 2007; Gaskins *et al*., 2012). Most of the cytokines present in the “cytokine storm” such as IL-6, IL-8 and TNF are inhibited by periovulatory levels of E_2_, while low levels of this hormone can increase inflammatory mediators, which could explain the pro-inflammatory states (Straub, 2007).

In pre-menopausal women, E_2_ has an anti-inflammatory effect, because high levels of this hormone, and consequently the action of E_2_ /ERα complex inhibits the transcriptional activity of NF-κB and downregulates the gene expression of pro-inflammatory cytokines, such as IL-6, IL-8 and TNF (Gaskins *et al*., 2012). In postmenopausal women low levels of this hormone are reported that can be supplemented with the use of hormone replacement therapy, especially with E_2_ (Giefing-Kröll *et al*., 2015), in this case, another study indicates that CD4+ monocytes and macrophages derived from monocytes deprived of E_2_ express higher levels of CD16, with significant increases in the production of TNF, IL-1β and IL-6 due to the absence of estrogen (Kramer *et al*., 2004).

In reproductive phase women, E_2_ in contraceptive methods was noted as a cellular immunity enhancer in HIV-infected patients (Hel *et al*., 2010) and in animal models, such as rats, when receiving high doses of E_2_ survival rate increased and lung cytokine production after influenza infection decreased (Robinson *et al*., 2011).

In *SARS-CoV-2* infection the activation of essential cellular proteins such as ACE2 and TMPRSS2 are key for cell recognition and virus entry, and both are highly expressed in lung tissue (Hoffmann *et al*., 2020). In an *in vitro* study conducted as a biological model for *SARS-CoV-2* infection and treatment with E_2_, VERO E6 cell line of monkey kidney, assessing ACE2 and TMPRSS2 mRNA levels indicated a negative regulation of mRNA levels of *TMPRSS2* when cells were pre-treated with this hormone. These data suggest that E_2_ may reduce *SARS-CoV-2* infection through TMPRSS2 inhibition, preventing viral dissemination and minimising ACE2 cleaving to increase the pathogen’s entry into the host cell (Lemes *et al*., 2021). To corroborate these results, *ACE2* and *TMPRSS2* activation in lung cells were evaluated in another *in vitro* study investigating the culture of human lung epithelial cells (A549) treated with E_2_. The results showed a negative regulation in *ACE2* and *TMPRSS2* mRNA levels, suggesting that decreased gene expression may be involved with the lower incidence of morbidity and mortality (Baristaite and Gurwitz, 2022). An important factor affecting gender differences in COVID-19 include not only androgen-mediated transcription of TMPRSS2, but also X-linked effects such as ACE2, the androgen receptor, and *TLR7* loci, all located on the X chromosome (Wambier and Goren, 2020). All these studies suggested E_2_ as adjuvant therapy and pointed out the need for experimental studies in animal models.

E_2_ is known to modulate the risk of cardiovascular diseases (CVDs) and still plays an important role in regulating RAAS expression and activity (Gallagher *et al*., 1999). The identification of ACE2 as a host cell receptor for *SARS-CoV-2* has drawn attention to the functions of this enzyme outside the domain of its role in modulating Ang II metabolism as part of RAS (Zhou *et al*., 2020). Activation of angiotensin II (Ang II) from the NF-κB pathway increases cytokine synthesis after SARS infection, while E_2_ can inhibit it, has great relevance for COVID-19 treatment strategies (Al-Lami *et al*., 2020).

Corroborating these findings, the E_2_/ERα complex inhibited NF-κB-mediated inflammatory response and cytokine production via lymphocytes, macrophages, and neutrophils (Biswas *et al*., 2005). CD14+ monocytes and macrophages derived from monocytes deprived of E_2_ express higher levels of CD16, with significant increases in the production of TNF, IL-1β and IL-6 due to the absence of E_2_ (Kramer *et al*., 2004). 

In summary, the accumulating evidence of a somewhat lower rate of COVID-19 severity in women needs further investigation. Large databases are being generated in response to the pandemic and need to be analysed for sex-related differences in the clinical presentation of the disease, age and medical history, including records of contraceptive use and menopausal hormone therapy (Groban *et al*., 2020).

## Search methodology

This study refers to a review of narrative literature, in which comprehensive bibliographic research was conducted in the scientific databases (PubMed and Scielo) until September 2022.

The research terms used were respiratory disease, Tuberculosis, *Mycobacterium tuberculosis*, COVID-19, *SARS-CoV-2*, gene regulation, immunogenetics, immune response, steroid hormones, estrogen, 17β-estradiol, and vitamin D_3_.

The requirements proposed for eligible articles in this review were: original data, reviews for conceptualization and English language. Studies with secondary data were not included. Therefore, our search results in the database were accessed, and relevant references were used for the proposed review.

## Final considerations

VD_3_ and E_2_ can act in several immunogenetics routes in order to restrain pathogen infections in the upper respiratory tract. The immune response is under hormonal control and gender differences, indicating that gene regulation differs in men and women and according to age. Herein we summarized gene pathways involved in general immune response in TB and COVID-19, two of the deadliest diseases worldwide, as well as the differences in genetic profiles of individuals. Women in childbearing age present a genetic and hormonal regulation to afford more controlled inflammatory and innate responses resulting in increased survival rate seen in TB and COVID-19. A lot has to be described, but considering how genetic and hormonal profiles influence infection resolution shed light onto the complexity and integrated system of general (populational) and specific (individual) immune response. 
